# Real-time adsorption and action of expansin on cellulose

**DOI:** 10.1186/s13068-018-1318-2

**Published:** 2018-11-22

**Authors:** Yuhao Duan, Yuanyuan Ma, Xudong Zhao, Renliang Huang, Rongxin Su, Wei Qi, Zhimin He

**Affiliations:** 1 State Key Laboratory of Chemical Engineering, Tianjin Key Laboratory of Membrane Science and Desalination Technology, School of Chemical Engineering and Technology, Tianjin University, Tianjin, 300072 China; 2 Biomass Conversion Laboratory of Tianjin University R&D Center for Petrochemical Technology, School of Chemical Engineering and Technology, Tianjin University, Tianjin, 300072 China; 3 School of Environmental Science and Engineering, Tianjin University, Tianjin, 300072 China; 4 Collaborative Innovation Center of Chemical Science and Engineering (Tianjin), Tianjin, 300072 China

**Keywords:** Expansin, Cellulose, Adsorption, QCM-D, Surfactant, Cellulase

## Abstract

**Background:**

Biological pretreatment is an environmentally safe method for disrupting recalcitrant structures of lignocellulose and thereby improving their hydrolysis efficiency. Expansin and expansin-like proteins act synergistically with cellulases during hydrolysis. A systematic analysis of the adsorption behavior and mechanism of action of expansin family proteins can provide a basis for the development of highly efficient pretreatment methods for cellulosic substrates using expansins.

**Results:**

Adsorption of *Bacillus subtilis* expansin (*Bs*EXLX1) onto cellulose film under different conditions was monitored in real time using a quartz crystal microbalance with dissipation. A model was established to describe the adsorption of *Bs*EXLX1 onto the film. High temperatures increased the initial adsorption rate while reducing the maximum amount of *Bs*EXLX1 adsorbed onto the cellulose. Non-ionic surfactants (polyethylene glycol 4000 and Tween 80) at low concentrations enhanced *Bs*EXLX1 adsorption; whereas, high concentrations had the opposite effect. However, sodium dodecyl sulfate inhibited adsorption at both low and high concentrations. We also investigated the structural changes of cellulose upon *Bs*EXLX1 adsorption and found that *Bs*EXLX1 adsorption decreased the crystallinity index, disrupted hydrogen bonding, and increased the surface area of cellulose, indicating greater accessibility of the substrate to the protein.

**Conclusions:**

These results increase our understanding of the interaction between expansin and cellulose, and provide evidence for expansin treatment as a promising strategy to enhance enzymatic hydrolysis of lignocellulose.

**Electronic supplementary material:**

The online version of this article (10.1186/s13068-018-1318-2) contains supplementary material, which is available to authorized users.

## Background

Bioconversion of lignocellulosic biomass into fuels and chemicals has attracted great interest over last few decades due to the increasing global energy demand, rural development, and environmental safety concerns [1]. Lignocellulosic biomass is mainly composed of cellulose, hemicellulose and lignin, while it also contains some minor components like ash and extractive in different samples [2, 3]. Due to the recalcitrant structure of lignocellulose, commercial development of biomass and bioenergy is limited by the performance of cellulolytic enzyme systems [4]. The accessible surface area of exposed cellulose is a particularly important factor in the regulation of the enzymatic hydrolysis process [5]. Therefore, a lot of methods have been developed to increase cellulase accessibility, which is evaluated by measuring characteristics such as specific surface area or pore volume [6, 7].

Effective binding between cellulose and cellulase is an essential step for enzymatic hydrolysis [8–10]. Physical or chemical pretreatments using dilute acid, organic solvent, etc. have been employed to increase cellulase accessibility, but many of these methods are not eco-friendly, since the used chemical agents such as acids and alkalis are likely to cause environmental pollution and some undesired byproducts [11–13]. Biological pretreatment is a more attractive alternative. As a biological pretreatment reagent, expansin has non-hydrolytic disruptive activity and can facilitate the cellulose hydrolysis [14, 15]. Expansins and expansin-like proteins were originally isolated from plants as cell wall-loosening factors [16], but their mechanisms of action are not fully understood, although the breakage of hydrogen bonds may be involved [17]. Expansins are modular proteins composed of two discrete domains connected by a short linker, which has very similar structure to that of cellulases. However, compared to the catalytic domain of glycoside hydrolase 45, the N-terminal domain of expansin lacks catalytic activity [18, 19]; the C-terminal domain (D2) resembles certain carbohydrate-binding modules. Both domains are required for the full cell wall-loosening activity of expansins [17, 20].

*Bacillus subtilis* (*Bs*) EXLX1 is a bacterial expansin that is a member of the EXLX family [21] and binds to the crystalline surface of cellulose through its D2 domain [22]. Soluble recombinant *Bs*EXLX1 has been expressed about 10 mg/l by *Escherichia coli*, proving it have cellulose-loosening effect [14]. Recently, *Bs*EXLX1 was expressed through *Pichia pastoris* system because it is one of the most effective and versatile systems for high-level expression of heterologous proteins about 860 mg/L, as well as several other advantages such as stable integration of foreign genes in the host genome, high-level secretion of foreign proteins and glycosylation modification, which improve the thermostability of proteins [23–25]. *Bs*EXLX1 has attracted interest for various applications owing to its strong binding capacity, synergistic action with cellulase, and ability to promote root colonization [14, 21]. It is known to bind to the crystalline surface of cellulose rather than to linear oligosaccharides [26]. In our previous study [23], we have proved that *Bs*EXLX1 displayed remarkable thermostability in a wide temperature range. Most importantly, *Bs*EXLX1 exhibited synergism in cellulose hydrolysis with endoglucanase, leading to twofold increase in the reducing sugar concentration when using expansin with 1 mg/g cellulose. To date, studies on *Bs*EXLX1 adsorption have been based on binding results after equilibrium, and real-time adsorption of expansins onto cellulose has not yet been investigated. Quartz crystal microbalance with dissipation (QCM-D) is an in situ surface-measuring technique that can be used to monitor adsorption behavior in real time, allowing the monitoring of changes in the mass and viscoelastic properties of a thin film surface. QCM-D has, therefore, been widely used to evaluate cellulase adsorption and activity on the cellulose surface [27, 28].

In this study, we investigated the *Bs*EXLX1 adsorption onto cellulose and activity by a combination of spectroscopy and electron microscopy approaches. The objectives of this study were (1) to monitor the real-time adsorption of *Bs*EXLX1 onto cellulose by QCM-D and the effects of protein concentration, temperature, and surfactants on this process; and (2) analyze changes in cellulose structure upon *Bs*EXLX1 treatment. The findings provide insight into the mechanism of expansin adsorption, binding, and action on cellulose that can broaden the applicability of these proteins to real-world problems.

## Results and discussion

### Production and purification of BsEXLX1

Recombinant *Bs*EXLX1 was expressed in *Pichia pastoris* and purified. The purified protein was verified by sodium dodecyl sulfate polyacrylamide gel electrophoresis (SDS-PAGE) (Additional file 1: Figure S1). The molecular weight of *Bs*EXLX1 was consistent with the predicted value of 26.1 kDa, but also showed bands of 30 and 35 kDa corresponding to the glycosylated form of the protein. After deglycosylated process, the molecular weight of *Bs*EXLX1 significantly decreased, implying that the recombinant proteins exhibited *N*-glycosylation (Additional file 1: Figure S2). The secondary structure prediction of *Bs*EXLX1 revealed that the protein has 8.21% α-helix, 39.13% β-sheet, 12.56% β-turn, and 40.1% random coil contents (Additional file 1: Figure S3A). To confirm these results, we examined the secondary structure of *Bs*EXLX1 by circular dichroism (CD) (Additional file 1: Figure S3B); purified *Bs*EXLX1 showed an obvious positive peak at 205 nm, indicating a β-sheet-rich structure.

### Adsorption of BsEXLX1 onto thin cellulose film

**Fig. 1:**
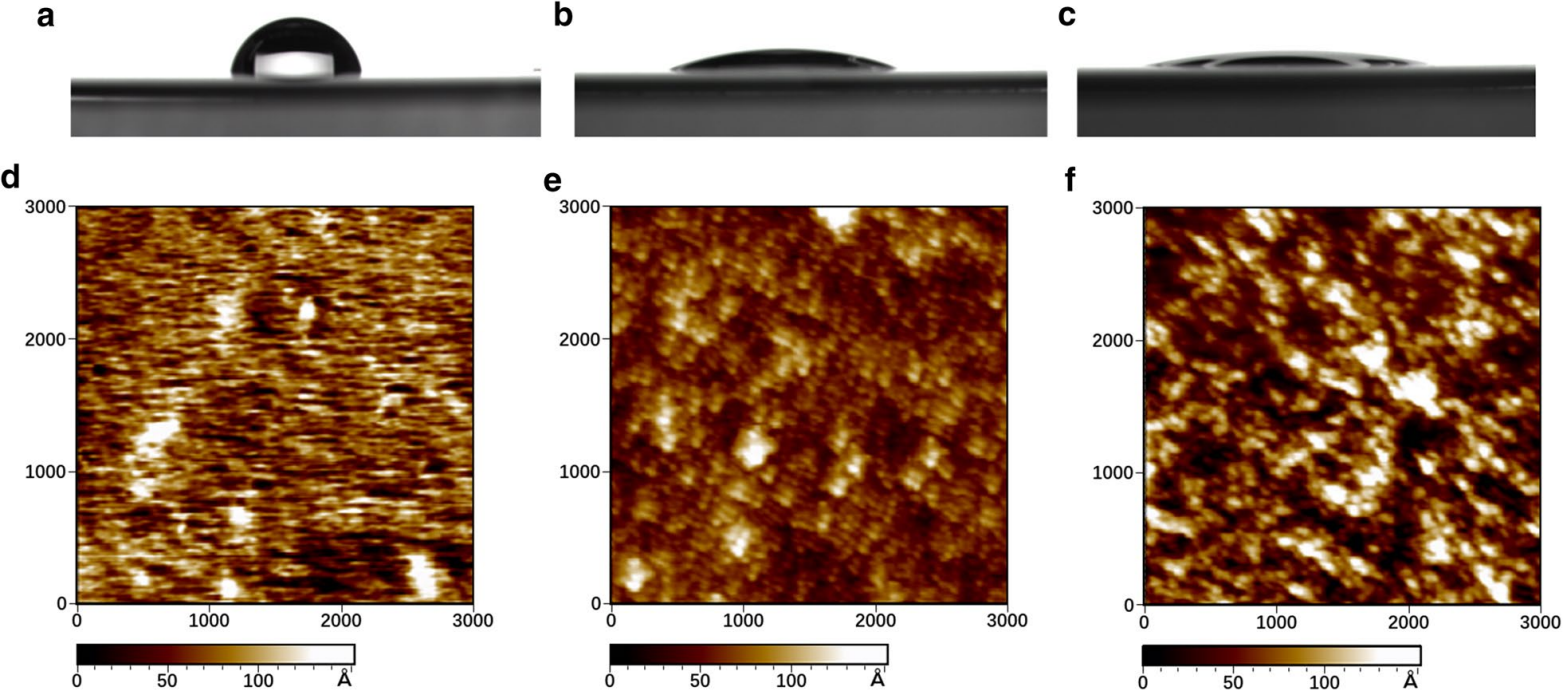
Wettability and morphology of cellulose film. CA and AFM images of gold (**a**, **d**); cellulose-coated surface in the dry state (**b**, **e**); and BsEXLX1-treated cellulose surface in the dry state (**c**, **f**)

The wettability and morphology of deposited cellulose film were investigated by contact angle (CA) measurement and atomic force microscopy (AFM), respectively (Fig. 1). The CA angle decreased from 17° (Fig. 1b) to about 7.3° (Fig. 1c) upon *Bs*EXLX1 treatment for 30 min, reflecting an increase in the hydrophilicity of cellulose. Hydrogen bonding was partly disrupted by expansin, leaving more free hydroxyls on cellulose to form hydrogen bonds with water, resulting in swelling after *Bs*EXLX1 injection. Compared to the gold surface of pure sensors (Fig. 1d), the cellulose film was smoother, with a root-mean-square RMS roughness of 33.28 Å (Fig. 1e). After 30-min adsorption of 50 ppm *Bs*EXLX1 on cellulose film, the RMS roughness of the surface increased to 55.92 Å due to the inhomogeneous adsorption (Fig. 1f), which was 70% higher than that of cellulose film.

**Fig. 2:**
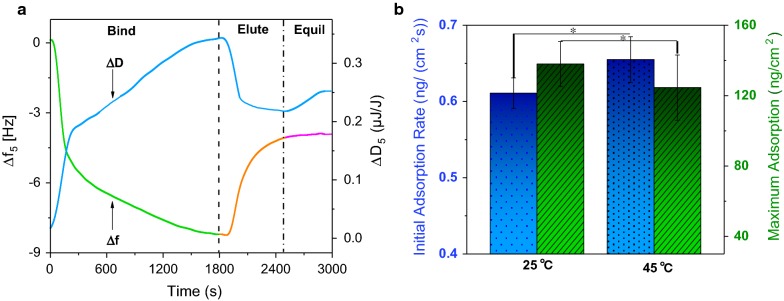
Adsorption of BsEXLX1 onto cellulose films. **a** Change in fifth overtone frequency and dissipation at 25 °C. *c* = 0.05 mg/ml, 50 mM sodium citrate buffer (pH 4.8). **b** Initial adsorption rate and maximum adsorption of BsEXLX1 at 25 °C and 45 °C

The adsorption of *Bs*EXLX1 onto cellulose film and the effects of concentration and temperature were investigated by QCM-D at pH 4.8. Adsorption was first examined with 50 ppm *Bs*EXLX1 at 25 °C. The protein was rapidly adsorbed in the first 3 min, with a decrease in frequency (about − 4.5 Hz) due to a large number of free adsorption sites; in the next 30 min, the frequency decreased by only 4.1 Hz (Fig. 2a). After rinsing the surface with sodium citrate buffer, some expansin was eluted and a new plateau was reached at about − 4.2 Hz. During the experiment, the change in dissipation (Δ*D*) increased to a maximum value before decreasing. Dissipation increased as the bulk of the film became more viscoelastic. In the initial stage, dissipation increased rapidly, reflecting fast protein adsorption (Fig. 2a). Adsorbed expansin can destroy the hydrogen bonds of cellulose [29], causing the cellulose film to swell and become less rigid. The decrease in the slopes of the change in frequency (Δ*f*) and Δ*D* curves occurred at 1400–1800 s, indicating that an adsorption equilibrium was reached. Buffer injection reduced the dissipation as some proteins were eluted. Interestingly, we observed a slight increase in dissipation after 2500 s, indicating a loosening of the cellulose structure caused by the sustained swelling effect of expansin.

Previous studies have shown the increased amount of free cellulases and enhanced cellulose hydrolysis at relative high temperatures [9, 30]. Limited by the temperature stabilization of QCM-D, we, therefore, examined the adsorption of *Bs*EXLX1 onto cellulose at 45 °C. The frequency data were converted into surface mass with the Sauerbrey equation (Fig. 2b). Compared to the behavior at 25 °C, less protein was adsorbed at 45 °C, indicating that *Bs*EXLX1 has relatively low combining energy with cellulose at high temperatures. Meanwhile, a faster initial adsorption rate was observed at 45 °C due to an increase in the internal energy of molecules. The effect of temperature on *Bs*EXLX1 adsorption was supported by analysis of variance at *P* < 0.05.

**Fig. 3:**
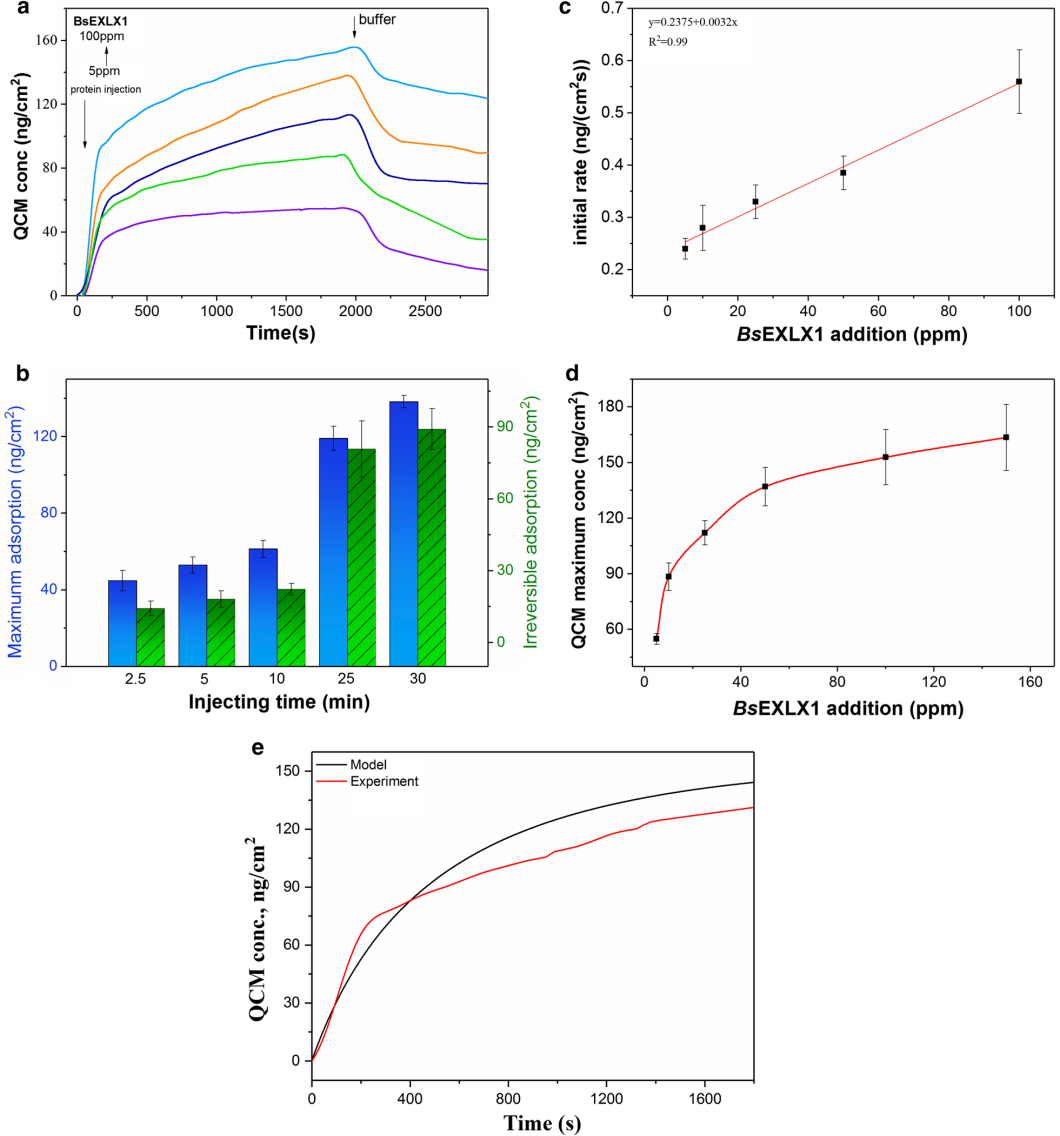
Transition model for BsEXLX1 adsorption onto cellulose. **a** Binding/washoff profiles of BsEXLX1 at different concentrations (25 °C, 100 μl/min). Protein concentrations from bottom to top are 5, 10, 25, 50, and 100 ppm. **b** Maximum and irreversible binding of 50 ppm BsEXLX1 with different injecting times. **c** Initial rate of BsEXLX1 adsorption to cellulose. The slope of the fit line is equal to the kinetic parameter *k*_A_·*Γ*_max_ = 0.2375 ng/(cm^2^ s ppm). *R*^2^ = 0.99. **d** The kinetic parameter *Γ*_max_ was determined from the adsorption of increasing concentrations of BsEXLX1 to the surface. The maximum surface concentration is shown (Bullet) vs. bulk expansion concentration, and reaches a near-maximal value of 163.53 ng/cm^2^. **e** Experimental binding isotherms at [*E*]_bulk_ = 50 ppm (red) compared to the prediction model (black)

To describe the adsorption process, a model was established by collecting adsorption and washoff data at various protein concentrations (Fig. 3a) and injection times (Fig. 3b). The initial adsorption rates of *Bs*EXLX1 were measured by varying protein concentrations from 5 to 100 ppm. Data from the first 3 min (fast adsorption stage) were used to calculate initial adsorption rate. Protein concentration and initial adsorption rate were linearly related (Fig. 3c), and the slope of the line was equal to the product of adsorption rate constant (*k*_A_) and maximum adsorption mass (*Γ*_max_), which was determined by applying increasing concentrations of *Bs*EXLX1 to the surface (Fig. 3d). When the concentration was increased from 100 to 150 ppm, *Bs*EXLX1 adsorption only changed by 9.5 ng/cm^2^; thus, the maximum value was determined as 163.53 ng/cm^2^.

The value of the parameters *k*_I_ and *k*_D_ was determined by the Eq. (7), and the multistage Runge–Kutta algorithm was used to calculate *k*_D_ + *k*_I_ at different least-squares method washoff times. The experimental and model curves are shown in Fig. 3e and model parameters are listed in Additional file 1: Table S2. The adsorption and irreversible binding rate constants were *k*_A,Cel7A_ = 0.28 s^−1^ and *k*_I,Cel7A_ = 1.7 s^−1^, respectively, for endoglucanase and *k*_A,Cel7B_ = 0.07 s^−1^ and k_I,Cel7B_ = 1.6 s^−1^, respectively, for exoglucanase, which are significantly higher than for *Bs*EXLX1 [31] and suggest a weaker binding force than cellulose. Although the results overestimate the adsorption (Fig. 3e) possibly due to the fact that *Γ*_max_ was not sufficiently accurate, the single-site transition model basically fits the observed adsorption behavior of *Bs*EXLX1 to cellulose.

### Effect of surfactants on expansin adsorption

Several non-ionic surfactants including Tween 20 [32], Tween 80 [33], Triton X100 [34] and Triton X114 [35] have been reported to improve the enzymatic hydrolysis of pretreated lignocellulosic biomass and pure cellulose. These are because the hydrophobic part of these surfactants can bind to the residual lignin of the substrates through hydrophobic interactions, thus preventing cellulase adsorption on lignin or protecting cellulase from denaturing. On the contrary, most ionic surfactants have an inhibitory effect for this process [36], while few of them could improve the efficiency of hydrolysis under certain conditions. Lin et al. [37] and Peng et al. [38] have reported that SDS-CTAB and dodecyltrimethylammonium bromide could be used to enhance this process respectively. In this work, we investigated the effects of three surfactants on the adsorption behavior of *Bs*EXLX1, including two non-ionic surfactants (Tween 80 and polyethylene glycol [PEG]4000) and an ionic surfactant (Sodium dodecyl sulfate [SDS]). Among these, Tween 80 and PEG4000 were usually used to enhance the cellulose hydrolysis due to its low cost and obvious effect, while SDS was one of the minority ionic surfactants could be applied to improve enzymatic hydrolysis in a certain concentration range [39, 40]. In these QCM-D experiments, the buffer was replaced with surfactant solution prepared with the same buffer. After maximum adsorption was achieved, the sodium citrate buffer was re-introduced to wash away loosely bound surfactant molecules from the substrate.

**Fig. 4:**
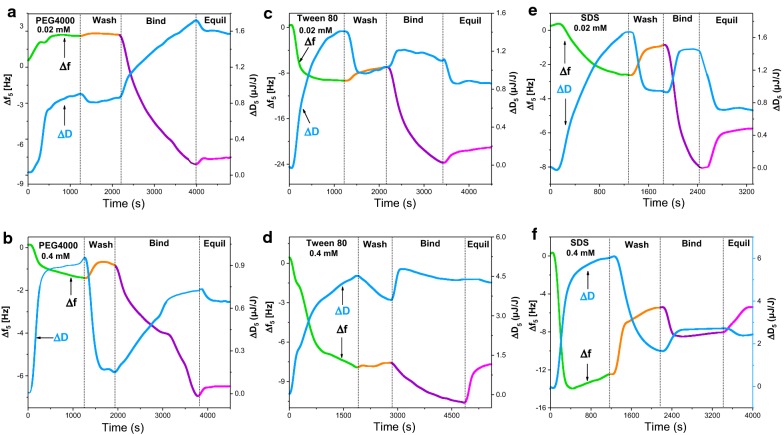
Changes in frequency and dissipation for adsorption of BsEXLX1 onto cellulose films with different surfactants. *c* = 0.05 mg/ml, 50 mM sodium citrate buffer (pH 4.8). PEG4000 (**a**, **b**), Tween 80 (**c**, **d**), and SDS (**e**, **f**) were used at 0.02 mM (**a**, **c**, **e**) and 0.4 mM (**b**, **d**, **f**). Maximum (**g**) and irreversible (**h**) adsorption of BsEXLX1 with different surfactants

The adsorption behavior of expansins in the presence of 0.02 mM non-ionic surfactants was evaluated (Fig. 4a, c). A 9.1-Hz decrease was observed for Tween 80, while a 2.3-Hz increase occurred for PEG4000 in the first 1200 s. Surfactants tended to adsorb onto surfaces, altering the surface and interfacial properties of the reaction system [41]; this was evidenced by the adsorption of Tween 80 onto the cellulose film, which is consistent with the increased dissipation. However, a flowing PEG4000 solution led to an increase in Δ*f* in the first 20 min (Fig. 4a), indicating a mass decrease that may have resulted from the detachment of loose, partially solubilized cellulose molecules from the chip, since PEG4000 has a relative long hydrophilic chain. The change in Δ*f* was associated with a gradual increase in Δ*D* (Fig. 4a), indicating that the substrate became less rigid after PEG4000 treatment.

*Bs*EXLX1 was injected after the washing step in which a baseline was reached by the buffer solution. The value of Δ*f* declined by 9.8 Hz and 15.8 Hz due to expansin adsorption onto PEG- and Tween 80-pretreated surfaces, respectively (Fig. 4a, c); these decreases were greater than that of expansin directly absorbed onto cellulose (Fig. 2a), indicating that 0.02 mM non-ionic surfactants can enhance binding between *Bs*EXLX1 and cellulose. Protein adsorption onto cellulose is a major contributor to hydrophobic and electrostatic interactions [27, 42]. Tween 80—which comprises PEGylated sorbitan as the hydrophilic headgroup linked to a monounsaturated, 18-carbon hydrophobic tail—was chosen to enhance the hydrophobicity and roughness of the substrate interface during the surfactant adsorption process [43]. On the contrary, after PEG4000 pretreatment, the cellulose surface became more hydrophilic due to the PEG4000 hydroxyl groups that prevented *Bs*EXLX1 adsorption. Meanwhile, reversible adsorption of *Bs*EXLX1 decreased after the system was eluted with acetate buffer, indicating that the stability of BsEXLX1 was improved by non-ionic surfactants, which is similar to the effect of Tween on cellulase. The change in dissipation also demonstrated that a protein film covered the cellulose.

The effect of non-ionic surfactants on *Bs*EXLX1 adsorption was further investigated using 0.4 mM PEG4000 and Tween 80. Injection of PEG4000 for 30 min resulted in a decrease in frequency of 1.3 Hz (Fig. 4b), indicating a mass increase. In contrast to adsorption at 0.02 mM, PEG4000 molecules showed a stronger tendency to escape from solution due to the high concentration difference. Two different mechanisms have been proposed for PEG adsorption—i.e., hydrogen bonding and hydrophobic interactions [40]. However, about 7.8-Hz adsorption was detected upon injection of 0.4 mM Tween 80 for 1800 s (Fig. 4d), reflecting a similar mass change as with the 0.02 mM solution due to steric hindrance and a limited number of adsorption sites.

Anionic and non-ionic surfactants reduced Cel7A adsorption to a lignocellulose substrate [39], which is similar to the effect of 0.4 mM surfactants on *Bs*EXLX1 adsorption. We observed only 6.1- and 2.9-Hz decreases (Fig. 4b, d), which are lower than the Δ*f* without surfactants and indicate an increase in ineffective adsorption. Non-productive interaction between cellulase and cellulose and cellulase solubilization were increased at certain surfactant concentrations due to inhibition of the adsorption process [35, 44]. As expansin-like proteins have a structure similar to that of cellulase—including near-identical carbohydrate-binding modules—non-productive adsorption was likely enhanced by the formation of surfactant–protein aggregates at a high concentration of surfactants. The excluded volume interaction is a mechanism by which surface-bound PEG polymers reduce protein adsorption onto surfaces [45]. Compared to PEG4000, Tween 80 preferentially occupies more sites and prevents the adsorption of proteins.

We also examined the effect of SDS, an ionic surfactant, on *Bs*EXLX1 adsorption onto cellulose (Fig. 4e, f). SDS molecules were absorbed onto cellulose through strong electrostatic and hydrophobic interactions, resulting in decreases in frequency of 2.6 and 13.9 Hz upon injection of 0.02 mM SDS for 1280 s and 0.4 mM SDS for 390 s, respectively. However, a 1.5-Hz increase was observed following injection of 0.4 mM SDS for 770 s, which may be related to the partial desorption of cellulose and SDS molecules from the substrate. In contrast to non-ionic surfactants, SDS inhibited *Bs*EXLX1 adsorption onto cellulose even at 0.02 mM, with a 7.2-Hz decrease observed after 20 min (Fig. 4e). *Bs*EXLX1 is a basic protein (pI > 9) that is positively charged in sodium citrate buffer (pH 4.8) [46]. However, SDS solution becomes more negatively charged with increasing concentrations. Therefore, when the *Bs*EXLX1 was injected, protein coagulation occurred due to strong electrostatic interactions with SDS, resulting in reduced protein absorption onto cellulose (Fig. 4f).

**Fig. 5:**
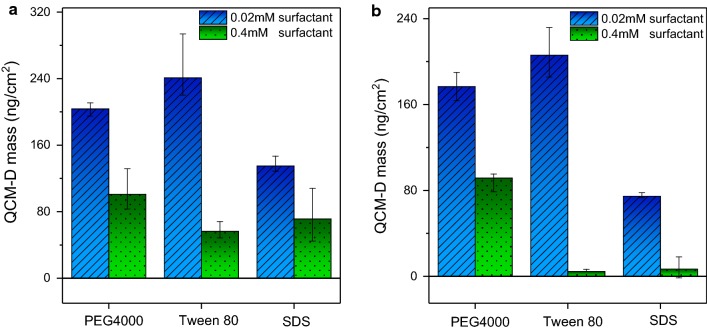
BsEXLX1 adsorption to cellulose with different surfactants. Maximum (**a**) and irreversible (**b**) adsorption of BsEXLX1 with different surfactants was calculated

Previous study [35] have investigated that non-ionic surfactants do not consistently improve the activity of cellulase, which showed a similar result with our research. Compared to the maximum (Fig. 5a) and irreversible (Fig. 5b) adsorption of *Bs*EXLX1 calculated with the Sauerbrey equation for different surfactants, Tween 80 and PEG4000 enhanced *Bs*EXLX1 adsorption at low concentrations and also decreased the reversible adsorption due to enhanced protein stability and changes in the interfacial properties. Compared to PEG4000, Tween 80 had a more potent inhibitory effect at high concentrations. However, no improvement of *Bs*EXLX1 adsorption was found in our research which has a difference effect of SDS on cellulose [40]. Low levels of adsorption were detected even with 0.02 mM SDS due to electrostatic interactions between the protein and anionic surfactant.

### Disruption of BsEXLX1 on cellulose

**Fig. 6:**
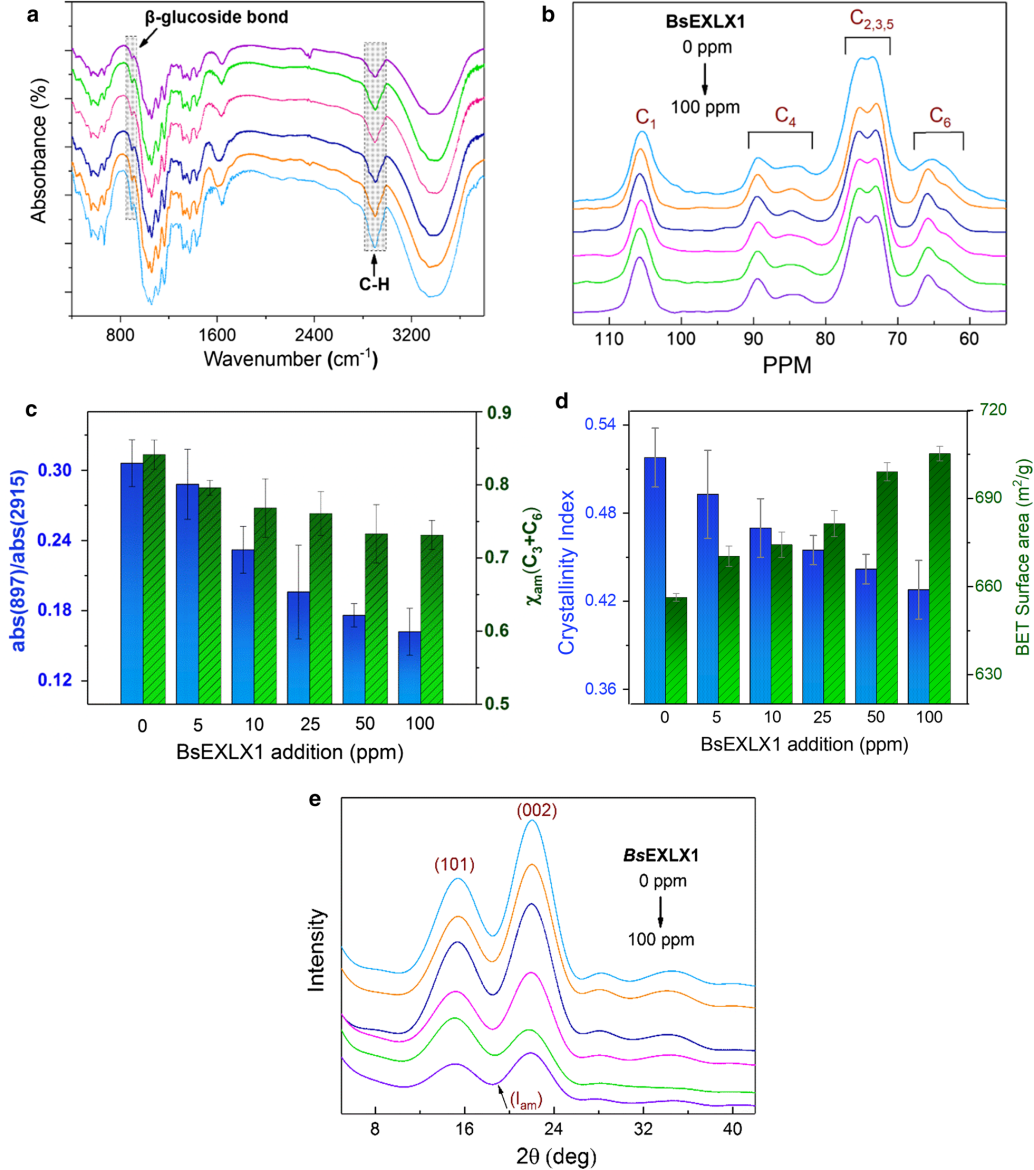
Disruption of BsEXLX1 on cellulose. Avicel PH-101 was incubated in 50 mM sodium acetate buffer (pH 4.8) with different concentrations (5, 10, 25, 50, and 100 ppm) of BsEXLX1 for 72 h at 45 °C with agitation (150 rpm). **a** FTIR spectra of BsEXLX1-treated and untreated Avicel PH-101. The bands at 897 and 2915 cm^−1^ were used for normalization. **b** Solid-state NMR spectra of BsEXLX11-treated and untreated Avicel PH-101. **c** Abs(897)/Abs(2915) in FTIR and solid-state NMR spectra. **d** Disruptive effect of BsEXLX1 at different concentrations on CrI and specific surface area. **e** XRD patterns of BsEXLX1-treated and untreated Avicel PH-101

To better understand the adsorption mechanism, it is important to investigate the structural changes of cellulose following *Bs*EXLX1 application. The disruption of hydrogen bonds by *Bs*EXLX1 was investigated by Fourier transform infrared (FTIR) spectroscopy and solid-state nuclear magnetic resonance (NMR). In the FTIR spectrum, the absorption peaks at 3419 and 2915 cm^−1^ were attributed to the stretching of hydroxyl groups and C-H stretching, respectively (Fig. 6a). Peaks at 1431 and 1375 cm^−1^ corresponded to CH_2_ stretching and CH bending, respectively. The peak at 897 cm^−1^ originated from β-glycosidic linkages associated with C_1_-H deformation and OH bending [47]. Compared to untreated Avicel, the peak at 897 cm^−1^ became weaker with increasing concentrations of *Bs*EXLX1, reflecting the partial destruction of intermolecular hydrogen bonds (Fig. 6a). The ^13^C NMR spectra of pure and pretreated Avicel showed absorption bands at 105, 90–80, and 65–60 ppm that were assigned to C_1_, C_4_, and C_6_, respectively, as well as C_2_, C_3_ and C_5_ absorption bands at 70–76 ppm (Fig. 6b). Changes in intramolecular hydrogen bonds showed the same trend as the FTIR spectroscopy results (Fig. 6c). Compared to pure Avicel, the relative value was decreased by 10% upon injection of 100 ppm *Bs*EXLX1.

X-ray diffraction (XRD) patterns for the original and pretreated cellulose samples (Fig. 6e) were used to determine the crystallinity index (CrI) upon *Bs*EXLX1 treatment. The index decreased with the intensity of the major scattering peak at about 22.3°. Upon treatment with 100 ppm *Bs*EXLX1, the crystallinity decreased to 0.428 as compared to 0.518 for pure Avicel (Fig. 6d). Thus, the decrease in CrI after the pretreatments was mainly due to the destruction of the cellulose crystalline structure. Changes in the shape and position of the XRD peak indicated the transformation of the original cellulose crystals into an amorphous substrate [48]. *Bs*EXLX1 caused the surface of Avicel to become rough and amorphic, as determined by scanning electron microscopy (SEM) (Additional file 1: Figure S4), which is consistent with previous findings for cellulose after expansin-like protein treatment [48].

### Accessibility

Accessibility is a key factor for enhancing cellulose hydrolysis. We measured the specific surface area and pore volume with the Brunauer–Emmett–Teller (BET) method based on nitrogen adsorption. Nitrogen passes readily though cell walls and its uptake provides a good estimate of total surface area, although it may not be directly related to enzyme accessibility due to size differences between nitrogen molecules and enzymes. With increasing *Bs*EXLX1 concentrations, the BET surface area increased from 656.38 to 698.97 m^2^/g (Fig. 6d), whereas, no changes in pore size distribution were observed (Additional file 1: Table S3). Thus, an increase in the specific area may be related to an increase in the number of pores, leading to greater accessibility of cellulose to *Bs*EXLX1.

## Conclusions

In summary, the adsorption and action of recombinant *Bs*EXLX1 on cellulose were investigated by various methods. The roughness and hydrophily of cellulose were increased by *Bs*EXLX1 treatment. The adsorption of *Bs*EXLX1 onto cellulose was investigated in detail by QCM-D, which revealed a rapid adsorption stage in the first 3 min followed by slow adsorption at 25 °C. The final amount of adsorbed protein decreased from 137.95 to 124.52 ng/cm^2^ when the temperature was increased to 45 °C. Injection of 0.02 mM Tween 80 and PEG4000 increased *Bs*EXLX1 absorption to 240.9 and 203.6 ng *Bs*EXLX1, respectively. However, adsorption was blocked in the presence of 0.4 mM surfactant due to an increase in ineffective adsorption. SDS enhanced *Bs*EXLX1 adsorption even at 0.02 mM due to strong electrostatic interaction between the protein and ionic surfactant. *Bs*EXLX1 disrupted the hydrogen bonding and crystallinity of Avicel, which can explain the increase in specific surface area from 656.379 to 698.97 m^2^/g of Avicel treated with 100 ppm *Bs*EXLX1. Thus, *Bs*EXLX1 can enhance the efficiency of cellulose hydrolysis for clean energy production and other applications.

## Materials and methods

### Materials

Avicel PH-101, 4-methylmorpholine-*N*-oxide, polydiallyldimethylammonium chloride (20% w/w), Tween 80, and PEG4000 were purchased from Sigma-Aldrich (Beijing, China). All other reagents such as phosphate-buffered saline, aqueous ammonia, sodium dodecyl sulfate, and sodium citrate were of analytical grade and were obtained from Aladdin Industrial Co. (Shanghai, China). Reagents were dissolved in Milli-Q water.

### Expression and purification of BsEXLX1

*Pichia pastoris* strain S-1 harboring the *Bs*EXLX1 gene cassette was used for *Bs*EXLX1 expression. Protein expression and purification were performed as previously described [23], with some modifications. The volume of the expression culture was increased from 30 to 300 ml, and *Bs*EXLX1 was purified by preparative chromatography (AKTA Purifier UPC 100) with a Ni-nitriloacetic acid His-bound resin column. The purified protein was separated by 12% SDS-PAGE and visualized by staining the gel with Coomassie brilliant blue G-250. In addition, we have also obtained the deglycosylated *Bs*EXLX1 according to our previous study [23]. The digested protein was assessed by a 12% SDS-PAGE gel to check the electrophoretic mobility shift.

### CD and modeling of reference data

Recombinant *Bs*EXLX1 protein solution was diluted in sodium citrate buffer to obtain a final concentration of 1 mg/ml. The secondary structure of the protein was evaluated by CD (JASCO, Osaka, Japan) at 25 °C within the range of 300–190 nm at a rate of 100 nm/min. Discovery Studio was used to predict the structure of *Bs*EXLX1 and generate a model of the protein.

### Preparation of cellulose film

Cellulose films were prepared on gold-coated QCM sensors (Västra Frölunda, Gothenburg, Sweden). The sensors were first cleaned by treatment with 25% ammonia solution/30% hydrogen peroxide/water (1:1:5, v/v/v) at 75 °C for 5 min followed by rinsing with Milli-Q water, drying with nitrogen, and ultraviolet (UV)-ozone plasma treatment for 15 min [27]. In the last step, incident UV light oxidizes any spurious adsorbed organic matter remaining on the surface of the sensor and also activates silanol groups required in later coating steps.

The QCM sensor was spin coated with cellulose solution as a uniform, thin film. Cellulose solution was prepared by dissolving microcrystalline cellulose (Avicel) in 50 wt% water/*N*-methylmorpholine-*N*-oxide at 115 °C. Dimethyl sulfoxide was added to adjust the concentration and viscosity of the polymer (0.05%) in the mixture.

The cellulose was applied to the sensor using a coater (Spin150-v3-NPP; Biolin Scientific, Linthicum Heights, MD, USA) spinning at 5000 rpm for 1 min. The cellulose-coated substrate was washed thoroughly with Milli-Q water and dried with nitrogen.

### Characterization of cellulose film

The morphology, roughness, and material distribution of cellulose film were characterized by AFM (Dimension Edge; Bruker, Saarbrücken, Germany). The images were scanned in tapping mode using a J-scanner and silicon cantilevers. At least two different films were prepared for each condition, and at least two different areas were analyzed on each. RMS roughness and Z sections in line profiles were determined from images, which were analyzed using Nanoscope v.V6.13 R1 software (Digital Instruments, Tonawanda, NY, USA).

The wettability of the films was determined using a CA measurement system (OCA15EC; Dataphysics, Filderstadt, Germany) by the sessile drop method with a drop volume of 3 μl. All measurements were performed with a minimum of four drops per surface at room temperature and an average value was calculated.

### QCM-D

The activity of *Bs*EXLX1 activity on the film was monitored in situ with a quartz crystal microbalance (QCM-D E1; Biolin Scientific). Immediately before measurement, sodium citrate buffer (pH 4.8) was introduced into the measuring chambers via a peristaltic pump at a flow rate of 0.1 ml/min. When the unit was filled with buffer solution, the frequency of vibration was continuously monitored until a stable signal was obtained. *Bs*EXLX1 protein in the same buffer solution was introduced into the chambers and Δ*f* was monitored. Following protein adsorption, buffer was again introduced into the chambers to wash off reversibly adsorbed protein. Measurements were performed at 25 °C unless stated otherwise. Δ*f* and Δ*D* for the fundamental frequency (5.0 MHz) and its overtones (*n* = 3, 5, 7, 9, 11, and 13) were monitored simultaneously and only the fifth overtone (*n* = 5) was used for data analysis. Frequency data were converted into surface mass values using the Sauerbrey equation:

1 $$ \Delta f = - \frac{{2f_{0}^{2} }}{{A\sqrt {\rho_{\text{q}} \mu_{\text{q}} } }}\Delta m, $$

where Δ*f* is the frequency shift measured by QCM; Δ*m* is the change in resonating mass associated with the sensor surface; *f*_0_ is the resonance frequency of the quartz crystal (5 MHz); *A* is the sensor surface area (about 1.539 cm^2^), *ρ*_q_ is the density of quartz (2.648 g/cm^3^), and *μ*_q_ is the shear modulus of quartz for AT-cut crystal (29.47 GPa). For a soft (i.e., viscoelastic) film, energy is dissipated in the film during the oscillation, thus the mass change on a soft (i.e., viscoelastic) film is not fully coupled to the oscillation and the Sauerbrey relation is not valid [49]. The damping (or dissipation) (*D*) is defined as

2 $$ \Delta D = \frac{{E_{\text{diss}} }}{{2{{\uppi }}E_{\text{stor}} }}, $$

where *E*_diss_ is the dissipated energy and *E*_stor_ is the total energy stored in the oscillator during one oscillation cycle.

### Kinetics of BsEXLX1 adsorption

The kinetics of *B*sEXLX1 adsorption was described by a single-site transition model [50]. Mass balance equations for *Bs*EXLX1 adsorbed to the surface were as follows:

3 $$ \frac{{{\text{d}}\varGamma_{E} }}{{{\text{d}}t}} = k_{A} [E]_{\text{bulk}} (\varGamma_{{\rm max} } - \varGamma_{\text{E}} - \varGamma_{i} ) - k_{\text{D}} \varGamma_{\text{E}} - k_{\text{I}} \varGamma_{\text{E}} , $$

4 $$ \frac{{d\varGamma_{\text{I}} }}{{{\text{d}}t}} = k_{I} \varGamma_{\text{E}} , $$

5 $$ \varGamma_{{\rm max} } = \varGamma_{E} + \varGamma_{I} + \varGamma_{O} , $$

where *Γ*_E_ and *Γ*_I_ are the mass-based surface concentrations of reversibly and irreversibly bound protein, respectively; *Γ*_O_ is the concentration of free sites on the surface; *Γ*_max_ is the maximum surface concentration; [*E*]_bulk_ is the bulk protein concentration; and *k*_A_, *k*_D_, and *k*_I_ are the adsorption, desorption, and irreversible adsorption rate constants, respectively.

To measure the adsorption rate constant *k*_A_, Eq. (5) was evaluated for *t* ≈ 0, and Eq. (2) was simplified to:

6 $$ \frac{{{\text{d}}\varGamma_{E} }}{{{\text{d}}t}} = k_{\text{A}} [E]_{\text{bulk}} \varGamma_{{\rm max} } $$

By measuring the adsorption at different protein concentrations, data from the first 3 min were used to calculate the initial adsorption rate, and a linear relationship between initial rate and protein concentration was obtained. The slope of this line was equal to *k*_A_*Γ*_max_.

To measure the kinetic rate constants for desorption and irreversible adsorption, the mass change for different contact times was measured by QCM-D. Reversibly bound protein was washed off with sodium citrate buffer until a stable frequency signal was recorded. The sum of kinetic parameters (*k*_D_ + *k*_I_) was obtained by Eq. (6) [31]:

7 $$ \frac{{\varGamma_{T} (t) - \varGamma_{{{\text{T}},\infty }} }}{{\varGamma_{{{\text{T}},0}} - \varGamma_{{{\text{T}},\infty }} }} = {\text{e}}^{{ - (k_{\text{D}} + k_{\text{I}} )(t - t_{0} )}} , $$

where t_0_ was set as the zero when washoff was initiated, and *k*_D_ + *k*_I_ did not change with contact time or [*E*]_bulk_. The parameters *k*_I_ and *k*_D_ were determined by numerically solving the mass balance equations during binding and washoff.

### Cellulose characterization

Avicel PH-101 was incubated in 50 mM sodium citrate buffer (pH 4.8) containing approximately 50 ppm purified recombinant *Bs*EXLX1 at 45 °C for 72 h. A control experiment without *Bs*EXLX1 was performed under the same conditions. The cellulose was washed by water and fully dried in an oven at 65 °C.

### FTIR spectroscopy

FTIR spectra were obtained using a spectrometer (AVATR 360; American Nicolet Corp., Mountain, WI, USA) in the 4000–400 cm^−1^ range with the KBr pellet technique. The degree of intermolecular hydrogen bonding was calculated with the formula [51]:

8 $$ {\text{Ar}}\left( { 8 9 7} \right) = {\text{abs}}\left( { 8 9 7} \right)/{\text{abs}}\left( { 2 9 1 5} \right) $$

The absolution at 897^−1^ and 2915^−1^ was attributed to β-glycosidic bonds and stretching vibrations of C–H, and abs(897) and abs(2915) corresponded to the peak areas at 897 cm^−1^, 2915 cm^−1^, respectively.

### XRD

Powder XRD patterns of Avicel were obtained using an X-ray diffractometer (D8 Advance; Bruker) with Cu Kα irradiation at a scan rate of 6° min^−1^ and step size of 0.02°. The following equation was used to estimate percent CrI in the sample [52]:

9 $$ {\text{CrI}} = \frac{{I_{002} - I_{\text{am}} }}{{I_{002} }} \times 100(\% ), $$

where *I*_002_ is the scattered intensity due to the crystalline portions of the sample and *I*_am_ is the scattered intensity due to the amorphous portion.

## ^13^C NMR

^13^C NMR was performed using a solid-state NMR spectroscopy instrument (InfinityPlus 300; Varian Medical Systems, Palo Alto, CA, USA). Intramolecular hydrogen bonding was calculated with the following formula [51]:

10 $$ \chi_{\text{am}} (C_{3} + C_{6} ) = \frac{1}{2} \left(\frac{{I_{\text{h}} (C_{4} )}}{{I_{\text{h}} (C_{4}) + I_{\text{I}} (C_{4} )}} + \frac{{I_{\text{h}} (C_{6} )}}{{I_{\text{h}} (C_{6} ) + I_{\text{I}} (C_{6} )}}\right) \times 100, $$

where *I*_h_ and *I*_I_ represent the high and low field areas, respectively, of the NMR.

### SEM

The morphology of Avicel treated with *Bs*EXLX1 or left untreated was examined by SEM (Hitachi, Tokyo, Japan; S-4800).

### BET method

Cellulose accessibility was evaluated according to the specific surface area [7] by the BET method based on nitrogen adsorption. Samples were degassed at 120 °C for 3 h and then cooled in the presence of nitrogen gas; the gas was allowed to condense on the surface and within the pores of cellulose. The BET surface area was determined using an ASAP 2460 analyzer (Micromeritics Instrument Corp., Norcross, GA, USA) at a temperature of 77.35 K. Pore volume determined with the Barrett, Joyner, and Halenda method [53].

## Additional file

**Additional file 1.** Additional tables and figures.

## Abbreviations

AFM: atomic force microscopy
BET: Brunauer–Emmett–Teller
BsEXLX1: *Bacillus subtilis* EXLX1
CA: contact angle
CD: circular dichroism
CrI: crystallinity index
FTIR: Fourier transform infrared
*k*_A_: adsorption rate constant
NMR: nuclear magnetic resonance
PEG4000: polyethylene glycol 4000
QCM-D: quartz crystal microbalance with dissipation
SDS-PAGE: sodium dodecyl sulfate polyacrylamide gel electrophoresis
SEM: scanning electron microscopy
SEM: scanning electron microscopy
UV: ultraviolet
XRD: X-ray diffraction
*Γ*_max_: maximum adsorption mass
Δ*D*: change in dissipation
Δ*f*: change in frequency
