# Supplementary material for: Real-time adsorption and action of expansin on cellulose
Source: Biotechnol Biofuels. 2018 Nov 22;11:317. doi: 10.1186/s13068-018-1318-2 (PMC6249958; doi:10.1186/s13068-018-1318-2)
Supplement: Supplementary file 1 — Additional file 1. Additional tables and figures. [file 13068_2018_1318_MOESM1_ESM.doc]

**Additional Information for**

**Real-time Adsorption and Action of Expansin on Cellulose**

Yuhao Duan,^1†^ Yuanyuan Ma,^2†^ Xudong Zhao,^1^ Renliang Huang,^3^ Rongxin Su,^1,d4^,* Wei Qi,^1,4^ and Zhimin He^1^

^1^ State Key Laboratory of Chemical Engineering, Tianjin Key Laboratory of Membrane Science and Desalination Technology, School of Chemical Engineering and Technology, Tianjin University, Tianjin 300072, China

^2^ Biomass Conversion Laboratory of Tianjin University R&D Center for Petrochemical Technology, School of Chemical Engineering and Technology, Tianjin University, Tianjin 300072, China

^3^ School of Environmental Science and Engineering, Tianjin University, Tianjin 300072, China

**^4^** Collaborative Innovation Center of Chemical Science and Engineering (Tianjin), Tianjin 300072, China

The email addresses of all authors are shown as follows.

Yuhao Duan: [dyhtju@tju.edu.cn](mailto:dyhtju@tju.edu.cn); Yuanyuan Ma: [myy@tju.edu.cn](mailto:myy@tju.edu.cn); Xudong Zhao: [xudong-z@qq.com](mailto:xudong-z@qq.com); Renliang Huang: [tjuhrl@tju.edu.cn](mailto:tjuhrl@tju.edu.cn); Rongxin Su: [surx@tju.edu.cn](mailto:surx@tju.edu.cn) ; Wei Qi: [qiwei@tju.edu.cn](mailto:qiwei@tju.edu.cn) ; Zhimin He: [enzyme@tju.edu.cn](mailto:enzyme@tju.edu.cn)

^†^Y. Duan and Y. Ma contributed equally to this work.

***Corresponding author.** E-mail: surx@tju.edu.cn (R. Su)

**Table S1** Water contact angles and RMS roughness of cellulose films before and after *Bs*EXLX1 treated

|  | Gold-coated | Cellulose-coated | *Bs*EXLX1 -treated |
| --- | --- | --- | --- |
| CA(°) | 77.8±0.2 | 16.5±1.05 | 7.5±0.6 |
| RMS roughness (Å) | 25.35±1.2 | 33.28±2.6 | 55.92±4.2 |

**Table S2 The parameters for the adsorption modeling.**

|  | k_A_(s^-1^ppm^-1^) | k_D_(s^-1^) | k_I_(s^-1^) |
| --- | --- | --- | --- |
| value | 2.9050×10^-4^ | 6.2784 | 9.128×10^-4^ |

**Table S3** Pore volume of Avicel treated or untreated by *Bs*EXLX1.

|  | pure | 5ppm | 10ppm | 25ppm | 50ppm | 100ppm |
| --- | --- | --- | --- | --- | --- | --- |
| BJH pore  volume(cm^3^/g) | 0.529±0.006 | 0.536±0.012 | 0.542±0.07 | 0.562±0.01 | 0.560±0.016 | 0.582±0.015 |


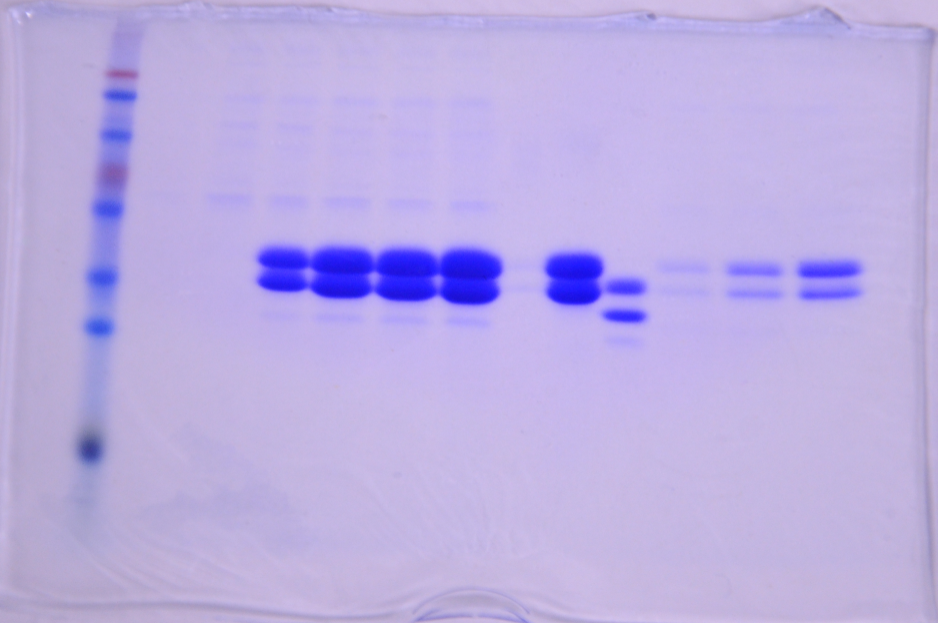


120k

100k

70k

50k

40k

30k

25k

**Figure S1.** SDS-PAGE analysis of *Bs*EXLX11: Lane 1. Mw standard; Lane 2-7. Correspond to day1 to day6; Lane 8. Blank I; Lane 9 purifcation of *Bs*EXLX1.


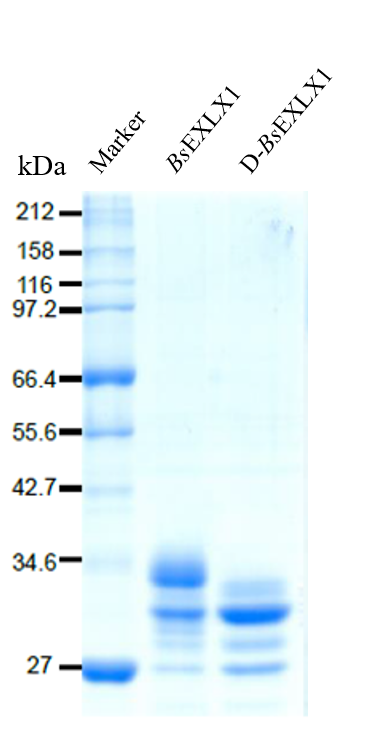


**Figure S2.** SDS-PAGE analysis of purified (Lane 2) and deglycosylated (Lane 3) *Bs*EXLX1.


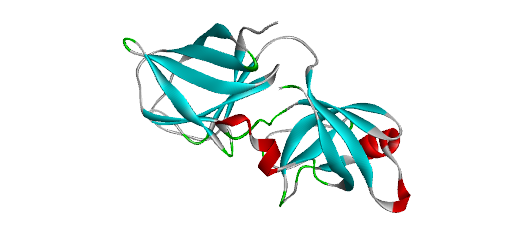


A)

**Figure S3.** CD spectra and homology model of *Bs*EXLX1. A) Modeling created by Discovery Studio. B) Net‑smoothed CD spectrum of *Bs*EXLX1.


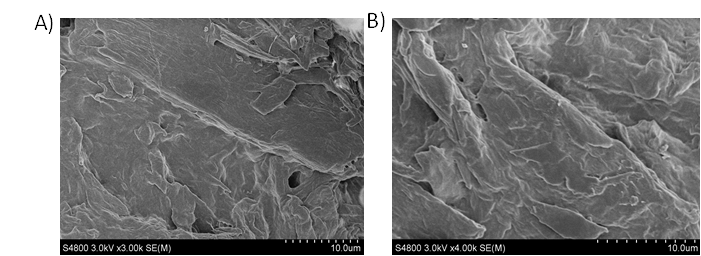


**Figure S4.** Disruptive activity of purifed *Bs*EXLX11 on Avicel. Scanning electron micrographs of Avicel in the A) untreated or B) treated by *Bs*EXLX1.
